# Optimal dsRNA Concentration for RNA Interference in Asian Citrus Psyllid

**DOI:** 10.3390/insects15010058

**Published:** 2024-01-12

**Authors:** Esmaeil Saberi, Mosharrof Mondal, Jorge R. Paredes-Montero, Kiran Nawaz, Judith K. Brown, Jawwad A. Qureshi

**Affiliations:** 1 Southwest Florida Research and Education Center, Department of Entomology and Nematology, IFAS, University of Florida, Immokalee, FL 34142, USA; esaberi@ufl.edu; 2 School of Plant Sciences, The University of Arizona, Tucson, AZ 85721, USA; mosharrof.mondal@gmail.com (M.M.); kirannawaz34@gmail.com (K.N.);; 3 RNAissance Ag, LLC, Saint Louis, MO 63132, USA; 4 Biology Department, Saginaw Valley State University, University Center, MI 48710, USA; jparedes@svsu.edu; 5 Facultad de Ciencias de la Vida, Escuela Superior Politécnica del Litoral, ESPOL, Campus Gustavo Galindo, Km 30.5 Vía Perimetral, Guayaquil EC090112, Ecuador

**Keywords:** *Diaphorina citri*, dsRNA biopesticide, gene knockdown, pest control, RNA interference

## Abstract

**Simple Summary:**

The Asian citrus psyllid is an insect pest of citrus trees that transmits the causal agent of huanglongbing disease. Double-stranded RNA molecules trigger RNA interference (RNAi), a naturally occurring process in eukaryotes that modulates the expression of genes involved in host innate immunity. Use of dsRNA biopesticides offers an alternative to traditional insecticides. In this study, the concentration of dsRNA required to achieve optimal knockdown and mortality for screening exogenous dsRNA by oral delivery was 200 ng/μL. Implementing this pre-established dsRNA regime for laboratory screening of ACP RNAi will enable high-throughput discovery of dsRNA targets and aid in evaluating RNAi biopesticide formulations. Establishing a working threshold can also prevent the use of excessive amounts of dsRNA, which is expensive, can negatively influence RNAi efficiency, and potentially trigger off-target effects.

**Abstract:**

The Asian citrus psyllid (ACP) is a citrus pest and insect vector of “*Candidatus* Liberibacter asiaticus”, the causal agent of citrus greening disease. Double-stranded RNA (dsRNA) biopesticides that trigger RNA interference (RNAi) offer an alternative to traditional insecticides. Standardized laboratory screening of dsRNA requires establishing the minimal effective concentration(s) that result in effective RNAi “penetrance” and trigger RNAi, resulting in one or more measurable phenotypes, herein, significant gene knockdown and the potential for mortality. In this study, knockdown was evaluated for a range of dsRNA concentrations of three ACP candidate genes, clathrin heavy chain (*CHC*), vacuolar ATPase subunit A (*vATPase-A*), and sucrose non-fermenting protein 7 (*Snf7*). Gene knockdown was quantified for ACP teneral adults and 3rd instar nymphs allowed a 48 h ingestion-access period (IAP) on 10, 50,100, 200, and 500 ng/µL dsRNA dissolved in 20% sucrose followed by a 5-day post-IAP on orange jasmine shoots. Significant gene knockdown (*p* < 0.05) in ACP third instar nymphs and adults ranged from 12–34% and 18–39%, 5 days post-IAP on dsRNA at 10–500 and 100–500 ng/µL, respectively. The threshold concentration beyond which no significant gene knockdown and adult mortality was observed post-48 h IAP and 10-day IAP, respectively, was determined as 200 ng/µL, a concentration indicative of optimal RNAi penetrance.

## 1. Introduction

The Asian citrus psyllid (ACP), *Diaphorina citri* Kuwayama (Hemiptera: Liviidae), is the insect vector of the fastidious bacterial plant pathogen “*Candidatus* (“*Ca*.”) Liberibacter asiaticus” (CLas), the causal agent of citrus greening disease, one of the most damaging diseases of citrus [1,2]. Management of citrus greening relies on conventional insecticides to reduce the populations of the ACP vector [3]. Widespread disease has resulted in increased use of insecticides and fertilizer and a greater risk of developing insecticide resistance in psyllid populations [4,5]. Double-stranded RNA (dsRNA) biopesticides exploit the naturally occurring RNAi pathway through which small interfering RNAs (siRNAs) facilitate gene silencing. Because sequence-specific dsRNAs can trigger gene knockdown in insects, RNAi offers a unique mode of action for pest management, while having minimal to no adverse effects on non-target species, including humans [6,7,8]. Specifically, double-stranded RNA molecules trigger RNAi, a naturally occurring process in eukaryotes that modulates the innate immune response and targets viral pathogens of an organism [9]. Gene silencing, or “knockdown of gene expression occurs when a dsRNA molecule targets a cognate messenger RNA (mRNA) for degradation, resulting in reduced gene expression [9,10]. Consequently, gene knockdown by RNAi offers a potentially unique approach for controlling hemipteran insects that are crop pests and vectors of “*Ca.* Liberibacter” spp. [11,12,13].

Recent studies have reported mortality and reduced fecundity following dsRNA-mediated knockdown of gene expression in ACP and the potato psyllid *Bactericera cockerelli* (Sulc.), a pest and vector of “*Ca.* Liberibacter solanacearum” [14,15,16,17,18]. Despite pioneering studies in which RNAi has been evaluated in diverse insects by delivering exogenous dsRNA or small interfering RNAs (by-products of degradation) [19] orally (sucrose or diet) or by dsRNA ingestion from plants expressing dsRNAs [12,20], effective environmental RNAi requires optimization at the species and/or family level. This is because the efficiency of dsRNAs delivered exogenously (by ingestion) to insects is governed by species-specific factors such as dsRNA dose and RNAi penetrance that can be influenced by gut pH and physiological stage [11]. Also, the relative effective concentration, or “dose”, of dsRNA required to effect phenotypic changes in a target organism, such as mortality, reduced fecundity, or interference with vector-mediated pathogen transmission, can vary by insect species, life stage, abundance of the target transcript, spatial and temporal gene expression, as well as dsRNA delivery method. Consequently, for each species the choice of target gene(s) and concentration of individual or “stacked” dsRNAs must be determined empirically [7,18,21,22,23]. While lower than optimal dsRNA concentrations may not trigger optimal RNAi [19], excessively high concentrations can lead to off-target or cytotoxic effects and/or over-saturation of the RNAi machinery that reduces RNAi efficiency [21,22]. For ACP, RNAi resulting in significant mortality requires dsRNA concentrations spanning 20–1000 ng/µL, 0.1–1000 ng/µL, and 0.3–500 ng/µL, depending on the delivery method such as ingestion from 20% sucrose solution or artificial diet [15,24,25,26,27], topical application [14,16,17,27,28,29], and plant-mediated delivery [7,11,15], respectively. For hemipterans, commonly used experimental delivery approaches for dsRNA are oral ingestion in 20% sucrose [15,24,25,26,27,28], diffusion after topical application into the hemolymph [14,16,17,27,28,29,30], and ingestion of detached leaves [7,11,15], or transgenic plant- or virus-vector (VIGS)-expressed dsRNA [13].

Characteristically, target selection in insects has focused on genes contributing to essential biological processes that have no negligible effects on non-target organisms [20,31,32]. In the potato psyllid, several gene targets have been identified that result in gene silencing and mortality or reduced fecundity [18,23], a tractable surrogate study system for evaluating gene target candidates singly or in groups (stacked) and selecting the most promising for ACP screening [23]. In ACP, gene silencing studies have demonstrated mortality, reduced fecundity and/or delayed development, and mortality, indicating the robustness of knockdown in ACP, and that phenotype may vary by gene target and/or delivery method [7,11,12,14,15,17,26,29,33,34].

The objective of this study was to determine the optimal concentration(s) of dsRNA required to achieve robust knockdown and mortality in ACP. The candidate genes, *CHC*, *vATPase-A*, and *Snf7*, selected for this study have been pre-screened for knockdown and mortality in potato psyllid [23]. The minimal and optimal effective dsRNA concentration(s), respectively, were evaluated for immature and adult ACP to identify those yielding the most robust RNAi penetrance. The relative knockdown was based on analysis by real-time quantitative RT-PCR amplification and mortality was assessed, post-IAP, for select genes previously reported to be susceptible to knockdown in other insects, including the potato psyllid [23].

## 2. Materials and Methods

### 2.1. Asian Citrus Psyllid Colony Establishment and Maintenance

The ACP colony was established with adult psyllids collected from citrus trees in an experimental orchard located at the Southwest Florida Research and Education Center (SWFREC) at the University of Florida, Immokalee, FL (lat. 26.42° N, long. 81.42° W) in 2006. Psyllids were reared on orange jasmine plants (*Murraya paniculata* (L.) Jack.) maintained by serial transfer (8–10 wks.) in an insect-proof cage (45 × 45 × 50 cm) (BioQuip, Rancho Dominguez, CA, USA). Colonies were maintained in a growth chamber held at 25 ± 2 °C with a 14:10 h (light: dark) photoperiod and relative humidity (RH) of 60–70%.

### 2.2. Design and Cloning of Double-Stranded RNA Targets and RNA Synthesis

#### 2.2.1. Selection of dsRNA Target Region and Off-Target Analysis

Three ACP genes, identified as homologs to promising gene targets identified for knockdown in potato psyllid [23], were evaluated at different dsRNA concentrations for the ability to result in significant gene knockdown and mortality. The gene targets were: clathrin heavy chain (*CHC*, GenBank Accession XM_026823405.1), a gene involved in the formation of clathrin-coated pits to mediate early endocytic uptake, regulation of nutrient uptake, and solute transport [35], vacuolar-type ATPase (*vATPase-A*, GenBank Accession XM_008471983.3) that encodes a proton pump–pH regulator associated with the cell membrane and governs many biological processes in cells and organelles, including regulating pH of intracellular organelles, receptor-mediated endocytosis, intracellular targeting of lysosomal enzymes, membrane trafficking of molecules, protein degradation, homeostasis of cytoplasmic pH, and coupled transport of small molecules [36,37], and sucrose non-fermenting protein 7 (*Snf7*, GenBank Accession XM_008471783.3), a protein involved in transport of proteins destined for degradation in the endosomal–autophagic pathway [38].

The template for dsRNA synthesis was a 200–250 bp fragment of the coding region for each gene. The optimal dsRNA size of <300 bp was selected based on a previous study [39] that evaluated different lengths of dsRNA on biological activity for the potato psyllid Ca. L. solanacearum (CLso) study system, a fast-track surrogate study system for ACP-CLas [39]. The off-target potential for each gene target was analyzed bioinformatically by considering all possible 21-mers that corresponded to siRNAs expected to result from dsRNA processing by dicer. The 21-mers were mapped against ACP transcriptome libraries assembled from the gut, salivary gland, adult whole-body, and nymph whole-body RNA-seq libraries [40] using Bowtie, allowing 0–1 mismatches per read, based on scanning for non-target hits in genome/transcriptome sequences available for arthropods commonly associated with citrus groves (citrus aphid, lacewing, ladybeetle) and humans [41]. The number of hits per transcript was calculated using BEDtools [42]. A hit to a non-target gene prompted additional searches of different 200–250 bp regions, and the pipeline was re-run. A 231 bp region of a firefly luciferase gene (Luc, AY535007.1:2532–4184), included as non-target control, was screened to identify potential off-target nucleotide matches using the pipeline (described above), producing the most optimal dsRNA regions of 147 bp, 153 bp, and 194 bp in size, for *CHC*, *vATPase-A*, and *Snf7*, respectively.

#### 2.2.2. Molecular Cloning of Double-Stranded RNA Targets in Psyllid and dsRNA Synthesis

The dsRNA target region of selected ACP genes was amplified from cDNA by polymerase chain reaction (PCR) with the respective gene-specific primer pairs (Table 1). Amplicons of the expected size were ligated into the pGEM-T Easy plasmid vector (Promega, Madison, WI, USA) using the TA-cloning system protocol and the manufacturer’s instructions. Ligation reactions were carried out using the pGEM^®^-T Easy Vector Kit (Promega, Madison, WI, USA). Transformation of *Escherichia coli* DH5α competent cells was carried out by the heat-shock method. To identify inserts of the expected size, three clones were screened by colony PCR amplification [43] using M13 primers (F-5′-TGTAAAACGACGGCCAGT-3′ and R-5′-AGGAAACAGCTATGACCATG-3′) and subjected to confirmatory bi-directional DNA sequencing (Sanger) (Eton Bioscience Inc., San Diego, CA, USA). Representative sequences have been submitted to the NCBI GenBank database and assigned the respective GenBank Accession numbers, XM_026823405.1, XM_008471983.3, and XM_008471783.3. Following sequence verification, amplicons were cloned into the MEGAscript plasmid vector downstream of the T7 promoter and used as template for dsRNA synthesis using the MEGAscript T7 Transcription Kit (Thermo Fisher Scientific, Waltham, MA, USA, Cat. No. AM1334), according to the manufacturer’s instructions. The dsRNA was synthesized by in vitro transcription using the MEGAscript T7 Transcription Kit (Thermo Fisher Scientific, Inc., Cat. No. AM1334), according to the manufacturer’s protocol [23]. The integrity of dsRNA was analyzed by agarose gel (1.2%) electrophoresis (TAE, pH 8.0) before and after each bioassay. The quality was determined based on optical density (O.D. 260/280) with a NanoDrop spectrophotometer and the DNA quantity was determined using the Qubit 4 instrument (ThermoFisher Scientific, Inc., Waltham, MA, USA).

#### 2.2.3. Ingestion-Access Period on Double-Stranded RNA

The dsRNA was delivered to ACP teneral adults (≤7 days) and 3rd instar nymphs by oral ingestion of dsRNA dissolved in 20 percent sucrose. Psyllids were starved for 4 h by holding them in a 35 by 10 mm plastic dish. The dish was surrounded by a narrow strip of moist cotton to maintain humidity and create a physical barrier that prevented psyllids from escaping. After the starvation period, the psyllids were transferred to a feeding chamber (15 and 30 adults and nymphs, respectively, per feeding chamber) containing dsRNAs at 10, 50, 100, 200, and 500 ng/µL, respectively, in 20% (*w*/*v*) sucrose solution with food dye (*v*/*v*) (purchased separately and mixed to final concentration of 0.1% green and 0.4% yellow) (McCormick & CO, Baltimore, MD, USA). Green food coloring was added to the sucrose to monitor psyllid feeding that results in excretion of green-colored honeydew. Adult dsRNA–sucrose ingestion was carried out according to a previously reported method [15]. Briefly, for adults, a layer of Parafilm M^®^ (Thomas Scientific (Swedesboro, NJ, USA, formerly Denville Scientific Inc.) was stretched over the surface of a 50 m Falcon tube. An aliquot of 20% sucrose solution (200 µL) containing green food coloring and dsRNA of each concentration tested, respectively, was pipetted onto the Parafilm and a second sheet of Parafilm was stretched over the first to create a sachet. Ingestion access by third instar nymphs was carried out using a previously reported method [44].

Double-distilled water or luciferase dsRNA (dsRNA-Luc) and food coloring were added to the 20% sucrose solution prepared in double-distilled water (ddH_2_O) and used as the negative and non-target controls, respectively. Feeding chambers were placed under fluorescent lights in an insectary maintained at room temperature with 60 to 70% relative humidity and a photoperiod of 14:10 h (light: dark). After psyllids were allowed a 48 h IAP on dsRNA–sucrose and/or ddH_2_O (negative control), they were transferred to orange jasmine shoots, placed in a Falcon tube containing ddH_2_O, and transferred to an insect-proof cage. The ACP-infested shoots were maintained for 5 d, post-dsRNA ingestion, and live psyllids were collected for RNA extraction. A 5-d post-dsRNA ingestion was chosen based on the RNAi persistence study [23] on the potato psyllid-CLso study system, as a surrogate for ACP-CLas. Each experiment consisted of three technical replicates per biological replicate (n = 3) that consisted of a cohort of 15 ACP adults or 30 third instar ACP nymphs per gene target. Total RNA was isolated from a pool of five ACP adults or 5th instar nymphs followed by cDNA synthesis.

#### 2.2.4. RNA Isolation, cDNA Synthesis, and Real-Time, Quantitative Reverse Transcriptase PCR Amplification

Total RNA was isolated from the pooled ACP cohorts using TRIzol^®^, according to the manufacturer’s instructions (Invitrogen, Carlsbad, CA, USA). According to the manufacturer’s instructions, residual genomic DNA was eliminated using a DNA-Free™ DNA removal kit (Invitrogen, Waltham, MA, USA). The quality of RNA was assessed using a NanoDrop ND 8000 spectrophotometer (NanoDrop Technologies, Wilmington, DE, USA). The cDNA synthesis was carried out using 2 ug total RNA in a 20 μL reaction with the high-capacity cDNA reverse transcription kit (Applied Biosystems, Carlsbad, CA, USA), according to the manufacturer’s protocol. Real-time, quantitative reverse transcriptase polymerase chain reaction (qRT-PCR) was carried out using TaqMan master mix (Applied Biosystems; Universal PCR Master Mix) primers and hydrolysis probes (Table 1) using the CFX96TM Real-Time PCR System (Bio-Rad Laboratories, Hercules, CA, USA). The qRT-PCRs were carried out in triplicate for each of three biological replicates, with water as the no-template negative experimental control. The Tm for primer–probe combinations was calculated using Benchling (https://benchling.com, accessed on 26 April 2021). The primer efficiencies were calculated using a series of 1:10 serial dilutions of template DNA. The Cq value for each serially diluted template reaction was plotted against the dilution power (1/10 = −1, 1/100 = −2, etc.) of the reaction. A line was fitted to the data, and the efficiency of the reaction was calculated using the formula $Efficiency\%=100\left(10^{\frac{-1}{slope}}-1\right)$ and ranged from 90–95% (Table 1).

Gene expression was normalized (1:1) in relation to non-target *luciferase* control using a previously published approach [18,23]. Statistical significance between the target gene and luciferase expression was determined by the CFX Maestro software v1.1 package (Bio-Rad Laboratories, Hercules, CA, USA). Statistical significance (*p* = 95%) between normalized expression of the gene of interest, relative to the non-target control, was analyzed using an unpaired *t*-test implemented in CFX Maestro 1.1. v. 4.1.2433.1219 software (Bio-Rad). Briefly, gene expression was quantified using the delta–delta quantification cycle method (ΔΔCq) [45], which normalizes expression with relation to a reference gene, calculated as fold change. The ACP ribosomal protein L5 (*RPL5*) gene was used as the internal reference gene (see reference [23] and those cited within). *RPL5* as a neutrally expressed gene for qRT-PCR analysis has been previously shown to be a stable reference gene for the potato psyllid *Bactericera cockerelli* (Sulc) [18,46], an ACP relative. The stability of *RPL5* reference gene expression was tested using the geNorm algorithm implemented in CFX Maestro software (Bio-Rad, Hercules, CA, USA) (Vandesompele et al., 2002). Validation of *RPL5* for qRT-PCR amplification of gene expression was carried out by comparing gene expression in ACP adult and nymphal stages post-ingestion of dsRNA-free sucrose and sucrose solution containing dsRNA-Luc at 100 ng/µL. Results of the analysis (Bio-Rad Maestro™ software) indicated that *RPL5* as the reference gene provided optimal stability of 0.82 and 1.22 [Ln(1/AvgM)] for ACP adults and nymphs, respectively. The results confirmed this gene was comparable for PoP and ACP, with respect to efficiency. The mean differences between knockdown of the target gene and ACP mortality at all concentrations were analyzed by ANOVA, and mean separation was based on Tukey’s honestly significant difference (HSD) at a 5% level of significance using InfoStat^®^ version 2020.

## 3. Results

### 3.1. Knockdown of Gene Expression for ACP Adults and 3rd Instar Nymphs, Post-dsRNA Ingestion-Access Period

#### 3.1.1. Relative Normalized Gene Expression of Target Genes in ACP Adults

**Figure 1:**
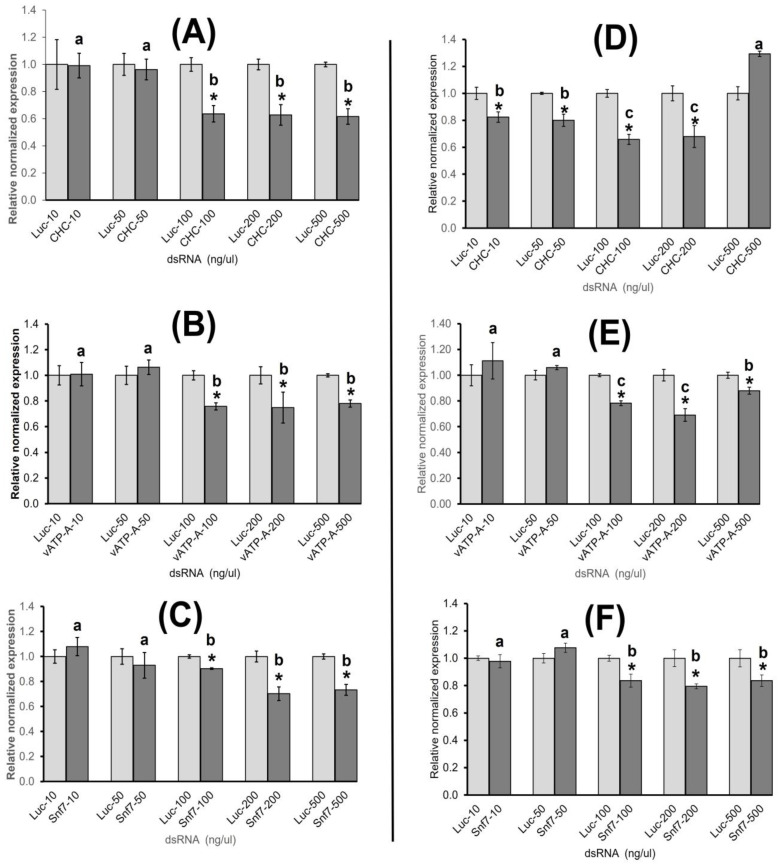
Gene knockdown by real-time quantitative PCR amplification in Asian citrus psyllid (ACP) post-ingestion-access period (IAP) with dsRNA at concentrations of 10, 50, 100, 200, and 500 ng/µL. Normalized relative expression of clathrin heavy chain (*CHC*), vacuolar ATPase subunit A (*vATPase-A*), and sucrose non-fermenting protein 7 (*Snf7*) in adults (**A**–**C**) and nymphs (**D**–**F**). Psyllids were given a 48 h IAP on 20% sucrose solution containing dsRNA. The qPCR amplification was carried out 5 days post-dsRNA IAP on young shoots of orange jasmine, *M. paniculata* plants. Significant differences in relative expression between test dsRNAs and luciferase dsRNA as the non-target control were evaluated for the same concentrations using three independent biological replicates of all three gene targets. Columns indicate the mean and standard error bar for each, respectively. The significant difference between a treatment and control at the same concentration analyzed by the Student’s *t*-test (*p*-value < 0.05) is indicated by an asterisk (*). Dark columns with different letters indicate statistically significant differences in gene-level expression rates between different dsRNA concentrations tested for the same gene (ANOVA with Tukey’s HSD test, *p*-value < 0.05).

Ingestion of dsRNA at concentrations of 100, 200, and 500 ng/µL by adults resulted in significant (*p* < 0.05) knockdown of *CHC* gene expression at 36.41, 37.24, and 38.37%, respectively, compared to the analogous dsRNA-Luc concentrations (Figure 1A). Expression of the *vATPase-A* gene was reduced by 24.19, 25.14, and 22.02% for dsRNA at 100, 200, and 500 ng/µL, respectively, compared to the analogous dsRNA-Luc concentrations (Figure 1B). Gene expression of *Snf7* was reduced by 18.46, 29.79, and 26.73% at the dsRNA concentrations of 100, 200, and 500 ng/µL, respectively, compared to the analogous dsRNA-Luc concentrations (Figure 1C). Significant differences in knockdown were not observed among the three highest dsRNA concentrations of 100, 200, and 500 ng/µL, while 10 and 50 ng/µL dsRNA did not result in statistically significant knockdown for the three genes, compared to the analogous dsRNA-Luc concentrations.

#### 3.1.2. Relative Normalized Gene Expression of Target Genes in ACP Nymphs

ACP nymphs allowed a 48 h IAP on dsRNA-CHC exhibited significant (*p* < 0.05) knockdown of *CHC* expression at 10, 50, 100, and 200 ng/µL dsRNA concentrations averaging 17.58%, 19.99%, 34.19%, and 32.00%, respectively, compared to the analogous dsRNA-Luc concentrations (Figure 1D). Knockdown was significantly higher at 100 and 200 ng/µL concentrations, compared to 10 and 50 ng/µL with no difference between the two former or two latter concentrations. In contrast, no significant knockdown of *CHC* expression was observed at a dsRNA concentration of 500 ng/µL (Figure 1D). Nymphs allowed a 48 h IAP on dsRNA-vATPase-A at dsRNA concentrations of 100, 200, and 500 ng/µL exhibited significant knockdown of *vATPase-A* averaging 21.74%, 30.95%, and 12.08%, respectively, compared to the analogous dsRNA-Luc concentrations (Figure 1E). Knockdown was higher for the dsRNA concentrations of 100 and 200 ng/µL than for 500 ng/µL, while no significant difference was observed between the dsRNA-vATPase-A concentrations 100 and 200 ng/µL.

Nymphal feeding of dsRNA-Snf7 at concentrations of 100, 200, and 500 ng/µL resulted in knockdown of *Snf7* at 16.40%, 20.52%, and 16.42%, respectively, with no differences between the three concentrations (Figure 1F). For *vATPase-A* and *Snf7*, dsRNA concentrations of 10 and 50 ng/µL did not result in statistically significant knockdown compared to the analogous dsRNA-Luc concentrations (Figure 1E,F).

Results demonstrated that the level of gene knockdown post-dsRNA ingestion was variable for the arbitrarily selected gene targets and ACP life stage. Knockdown was significantly higher for *CHC* at 36.41% and 34.19% compared to *vATPase-A* at 24.19% and 21.74% and *Snf7* at 18.46 and 16.40% for 100 ng/µL (*p* < 0.05) for the ACP adult and nymphal stages, respectively, however, no significant difference in knockdown was observed between the dsRNA-vATPase-A and -Snf7 treatments. For ACP adults, at a concentration of 200 ng/µL, gene knockdown was not significantly different for any gene target tested. In contrast, ACP nymphs exhibited significantly greater knockdown with dsRNA-*CHC* (32.00%), followed by dsRNA-vATPase-A (30.95%) and then dsRNA-Snf7 (20.52%) (*p* < 0.05). Adults given an IAP on 500 ng/µL dsRNA showed significantly higher knockdown for dsRNA-*CHC* (38.37%). However, no significant difference in knockdown was observed for the other two genes. In contrast, ACP nymphs exhibited the greatest significant knockdown post-IAP on dsRNA-Snf7 (16.42%) at 500 ng/ul, followed by dsRNA-vATPase-A (12.08%). No knockdown was detected post-*CHC* ingestion on dsRNA-CHC at a concentration of 500 ng/µL.

**Figure 2:**
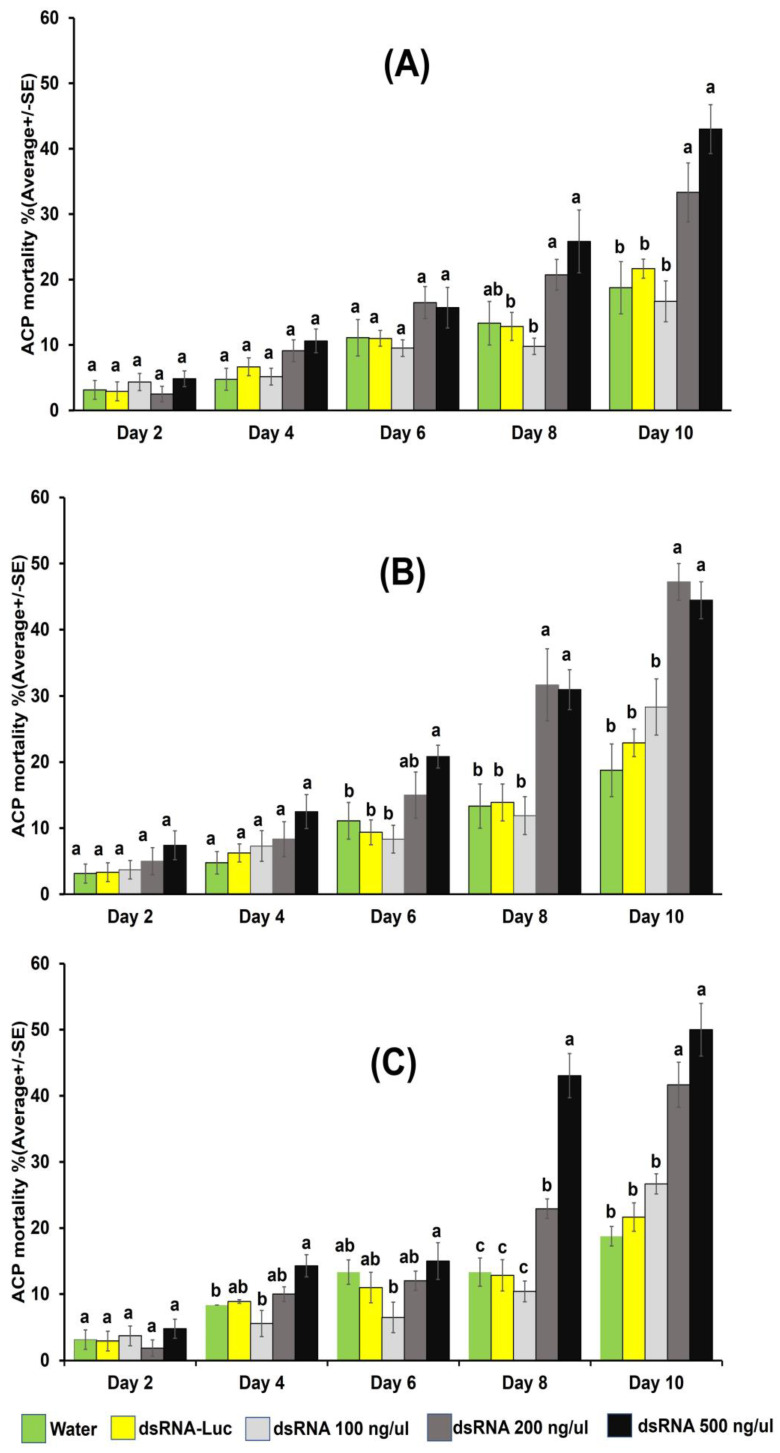
Mortality of *Diaphorina citri* adults allowed a 10-day continuous ingestion-access period on 20% sucrose containing dsRNA-clathrin heavy chain (*CHC*) (**A**), dsRNA-vacuolar ATPase subunit A (vATPase-A) (**B**), and dsRNA-sucrose non-fermenting protein 7 (Snf7) (**C**), at concentrations of 100, 200, and 500 ng/µL. RNase-free water and dsRNA-luciferase were included as the non-treatment and non-target control, respectively, and 20% sucrose was included as the negative control. Columns indicate the mean and standard error bar for each, respectively. Columns sharing the same letter showed no significant difference in mean psyllid mortality between those treatments at the same time point (ANOVA with Tukey’s HSD test, *p*-value < 0.05).

For the 48 h dsRNA IAP on 10, 50,100, 200, and 500 ng/µL in 20% sucrose solution, mortality of ACP adults and 3rd instar nymphs was tabulated 5 days post-ingestion-access period on orange jasmine shoots. Mortality of treated psyllids was not significantly different from the controls at any dsRNA concentration evaluated (Figure S1, data for adults). In contrast, ACP adult mortality was observed in a follow-up experiment using a continuous 10 d IAP on the dsRNA molecules at concentrations that resulted in significant gene knockdown in the initial experiments (Figure 2).

The analogous mortality studies with ACP 3rd instar nymphs could not be carried out because nymphs do not survive beyond the 2 d IAP on 20% sucrose, and sucrose does not provide sufficient nutrition to sustain growth or for molting to the adult stage to occur.

### 3.2. Mortality of Psyllid Adults through 10-Day Continuous Ingestion-Access Period on dsRNA Concentrations

The effect of RNAi on ACP adult mortality was analyzed by allowing adults a 10 d continuous IAP on dsRNA molecules in 20% sucrose solution [15]. Adults exhibited different rates of mortality following a 10-day IAP on dsRNA-vATPase-A, -CHC, and -Snf7 at concentrations of 100, 200, and 500 ng/µL (Figure 2). On day 10, mortality averaged 18.8 and 23% in adults feeding on sucrose alone or on dsRNA-luciferase at 200 ng/µL, the negative and non-target control, respectively. Knockdown of the gene-specific dsRNAs at 200 and 500 ng/µL resulted in a significant increase in mortality compared to the dsRNA-luciferase control by day 10 for the three target genes. However, significant mortality post-IAP was not observed for any of the three dsRNAs at 100 ng/µL, compared to the control (Figure 2).

The dsRNA-CHC at 200 and 500 ng/μL caused 20.74 and 25.83% mortality eight days post-IAP, respectively, and 33.33 and 43.00% after 10 days, respectively (Figure 2A). The dsRNA-vATPase-A at 200 ng/µL resulted in an average mortality of 31.67% and 47.22% at 8 and 10 days post-IAP, respectively, whereas ingestion of 500 ng/µL resulted in 30.95 and 44.44% mortality, respectively (Figure 2B). The earliest mortality of 20.83% was observed on day 6 psot-IAP on dsRNA-vATPase-A at a concentration of 500 ng/µL (Figure 2B). Significant ACP mortality was observed 8 and 10 days post-IAP on dsRNA-Snf7 at 200 ng/µL (22.93% and 41.67%, respectively) and 500 ng/µL (43.06% and 50.00%) (Figure 2C). No significant difference was observed in mortality between the dsRNA concentration of 200 and 500 ng/µL, respectively, for any of the gene-specific dsRNAs at 8 or 10 days IAP, except for dsRNA-Snf7, at eight days post-IAP, which resulted in greater mortality for 500 ng/µL, compared to mortality at 200 ng/µL (Figure 2C).

## 4. Discussion

There is growing interest in using RNAi biopesticides for insect pest control in agricultural crops, including for managing insect vectors of plant pathogens, such as ACPs and the potato psyllid [12,47]. Development of RNAi for insect pest management at the field level requires case-by-case studies to optimize the specificity and efficiency of RNAi, or “penetrance”, in the target organism to achieve the phenotype of interest, such as mortality, reduced fecundity, or lowered rates of transmission, as well as practical, easy-to-use, and effective delivery methods [12,47]. The optimal “dose” of dsRNA required to trigger an RNAi response in hemipteran insects is known to vary among insect species and may differ at the genus or family level and by developmental stage within a species, the persistence of dsRNA or siRNA molecules, and/or delivery method [48]. To achieve a robust RNAi response by oral delivery of exogenous dsRNAs, five dsRNA concentrations were evaluated in silencing the target ACP genes in both nymphal and adult stages. The results of this study indicate that ACPsare moderately sensitive to RNAi, based on significant gene knockdown of the three candidate target genes tested with the relatively low concentrations of dsRNA, of 10 to 100 ng/µL, which is consistent with previously published results [14,17,23,49]. However, a positive correlation was observed between gene knockdown and dsRNA concentration for the remaining dsRNA concentrations. In this and previous studies, a direct correlation between concentration and RNAi potency has been documented, with most showing a positive association between dsRNA concentration and robustness of the RNAi response [14,17,25,34,50]. Here, ingestion of dsRNA at 10–500 and 100–500 ng/µL resulted in a significant reduction in gene expression, ranging from 12–34% and 18–39% for ACP nymphs and adults, respectively.

The results indicated that the optimal effective concentrations for significant gene knockdown and mortality in ACP were 100 and 200 ng/µL, respectively. Also, concentrations of dsRNA ranging from 30–500 ng/µL have been shown to provide detectable to robust RNAi in ACP depending on the delivery method [12]. Psyllid uptake of dsRNA at 30–100 ng/µL [14,25] and, specifically, of dsRNA-cytochrome P450 at 50 ng/µL by topical application was sufficient to achieve knockdown that led to significant mortality and/or phenotypic abnormalities that resulted in death [29]. Topical application of dsRNA targeting DcMP20 [34] and DcSuh [17] at concentrations of 75 and 500 ng/µL, respectively, resulting in dsRNA diffusion in the hemolymph, provided the most optimal gene knockdown and mortality compared to the other concentrations tested. By comparison, oral ingestion of dsRNA at concentrations of 100 ng/µL [50] and 200 ng/µL [15] was the most optimal for gene knockdown and low rates of survival in the potato psyllid, an observation that is consistent with the results reported here. Further, increasing the dsRNA concentration to 500 ng/µL resulted in no significant negative effect on RNAi efficiency (Figure 1), a finding similar to previous studies that reported a non-direct correlation between a high concentration of dsRNA and RNAi response in ACPsand other insects [11,14,34,51,52]. Topical application of 100 ng of abnormal wing disc (AWD) dsRNA resulted in significant mortality in fifth instar ACP nymphs; however, increasing the dose to 10 ug did not cause increased mortality [14]. A similar pattern was observed for dsRNA arginine kinase (dsRNA-AK), when applications to citrus flush at 10 ug and 100 ng caused 53% and 56% mortality, respectively [11]. Likewise, Galdeano et al. (2017) showed that dsRNA concentrations greater than 200 ng/µL to target cathepsin D (CD), chitin synthase (CS), and inhibitor of apoptosis gene (IA) were not effective in increasing gene silencing or mortality of ACP. Indeed, a decrease in lethality was observed with concentrations of 500 and 1000 ng/µL of dsRNA homologous to CD or IA, respectively, compared with 200 ng/µL [15]. Therefore, in dsRNA studies reported for ACP, thus far, a saturation effect of RNAi has been observed when dsRNA exceeds a certain threshold [31,34,51]. Oversaturation of the components involved in the siRNA and RNAi response can interfere with the miRNA pathway leading to lethality or phenotypes related to the loss of miRNA function [22].

The relationship between dsRNA concentration and gene silencing (knockdown) in ACPs was influenced both by the specific target gene and life stage, e.g., adult or immature life stage [47], and results are consistent with those of previous studies in which the efficiency of gene silencing differed by target gene and by life stage (Figure 1). Greater variability of RNAi responses was observed for ACP gene targets evaluated in the immature compared to the adult stage. An IAP on the lowest concentration of dsRNA-CHC, 10 ng/µL, by ACP nymphs showed significantly reduced *CHC* gene expression while no significant knockdown was observed with this concentration for ACP adults (Figure 1A,D). However, increasing the concentration of dsRNA-CHC and dsRNA-vATPase-A to 500 ng/µL for nymphs had a negative effect on RNAi efficiency, compared to ACP adults for both genes (Figure 1D,E). Determining the most suitable life stage(s) to effect dsRNA treatments for lab screening and for field use has become important for the next steps in evaluating RNAi targets for effective knockdown and manifestation of desired phenotypes in ACPs [14]. Targeting genes vital to the different life stages of insects would be expected to affect their biological function, potentially specific to a particularly vulnerable developmental stage [53,54]. Silencing has been observed in different specific stages and/or instars of insects [52,53]. For example, the application of dsRNA-sucrose hydrolase homolog (DcSuh) in ACP adults resulted in reduced gene expression of DcSuh, but to a lesser extent (~40%) than that observed in nymphs (~90%) and newly emerged adults (~80%) [17]. This may be due to the nature of the targeted gene and its function(s) and/or to the different susceptibilities among ACP life stages to RNAi and the suitability of the delivery method [14,15,55].

The results indicated that among the targets tested here, dsRNA-CHC triggered the most robust silencing based on gene knockdown. However, silencing of dsRNA-Snf7 produced the greatest mortality in ACP adults, while dsRNA-vATPase-A and dsRNA-CHC exhibited intermediate and the lowest mortality, respectively. Previous studies with ACPs have reported an inconsistent relationship between RNAi-mediated gene silencing and mortality [17,56] an outcome that has also been observed for other hemipterans [56], especially for oral delivery of dsRNA, which relies on direct interactions between exogenous dsRNAs and the midgut [57,58,59]. Silencing of the sucrose hydrolase gene (DcSuh) in nymphs and adults resulted in reduced sucrose hydrolase activity with no concomitantly significant mortality [17]. Ingestion of dsRNA-calreticulin (DcCRT) and -laccase (DcLAC) by ACPs from *M. paniculata* leaflets triggered significant gene knockdown but no discernable negative effect on psyllid survival [56]. Non-lethal phenotypes in hemipteran insects post-dsRNA ingestion, may occur despite evidence of gene knockdown. For example, when *Myzus persicae* (Sulzer, 1776) adults were allowed to feed on tobacco plants expressing siRNAs corresponding to the *hunchback* gene, although robust gene knockdown was observed, no detectable mortality was evident [57]. In general, the efficiency of dsRNA for effective gene silencing that results in lethality (mortality) is directly associated with the particular gene target, as well as the specific function(s) of the target gene and its involvement in one or more essential pathways [7,22,58]. The range of knockdown efficiency observed in this study was 12–39% less than previously reported in ACPs [7,11,14,24,34,50]. One or more factors may explain the lower than expected RNAi efficiency and relative gene knockdown, among which could involve degradation of dsRNA by gut enzymes [7,60], insufficient siRNA amplification and/or less than robust systemic spread [61], the method of dsRNA delivery, and/or the selection of a non-responsive gene target used for optimal dose determination [22,62]. The ACP genome encodes dsRNase homologs [7,12] that have been reported to degrade exogenous dsRNA in other insects by acting rapidly on dsRNAs upon uptake into the gut [12]. Consequently, to achieve the most robust gene silencing in insects, the dsRNA concentration administered must be consistent with the threshold required for optimal penetrance to compensate for degradation or other deleterious factors that may factor into less than optimal outcomes when exogenous dsRNAs are exposed to the gut contents or other compartments in the body, upon delivery [12,34].

These results have shown that triggering RNAi by gene knockdown of one or more of the test targets evaluated here resulted in substantial ACP mortality. However, the “dose” required to trigger silencing with concomitant mortality was target dependent and apparently influenced by the duration of dsRNA exposure. After a ten-day continuous IAP by ACP adults on dsRNAs at a concentration of 200 ng/µL, mortality was observed post-IAP for all three targeted genes. In contrast, ACP adults receiving a 2-day IAP on dsRNA-vATPase-A and dsRNA-Snf7, regardless of concentration, showed no significant mortality even 5 days post-IAP, despite gene knockdown. These results suggest that the duration of dsRNA exposure and the “post-exposure” period may be critical for achieving significant mortality or other detectable phenotypes by RNAi [47,56,63]. Consequently, transgene expression or other means of deployment of dsRNA in planta will likely be necessary to achieve an optimal concentration of continuously available dsRNA that persists in the vascular tissue, in particular, the phloem where psyllids feed. Alternatively, stabilizing dsRNA for foliar or soil applications with time-release technology and/or combined with translaminar adjuvants and surfactants [39] may offer alternative solutions.

Nonetheless, the experiments have shown that *CHC* and possibly *vATPase-A* and *Snf7* are promising RNAi targets for ACPs, particularly when stacked with dsRNAs that target complementary phenotypes of interest [18,23,33]. This rationale is based on knowledge that the targets evaluated for gene silencing in this study are recognized to be essential for physiological processes, including receptor-mediated endocytosis [64,65], intracellular membrane trafficking [64,65], intracellular membrane trafficking [35], and protein degradation [38] and developmental processes including oocyte formation [35,66,67], molting [68], and pupation [69], and, consequently, have been evaluated as potential targets for triggering RNAi in other insect pests and vector species [32,56,68,69,70,71].

## 5. Conclusions

This report describes the first systematic evaluation of dsRNA concentration and the statistically significant effects of different concentrations on gene knockdown compared to the non-target experimental control, luciferase, reflective of the potential saturation threshold for RNAi penetrance in ACPs. This study has facilitated the establishment of the optimal concentration of exogenous dsRNA delivery, which was determined as the concentration of 200 ng/μL, which results in a measurable mortality with significant gene knockdown. Concentrations less or greater than 200 ng/μL did not yield optimal mortality and/or result in increased knockdown and could result in direct or indirect toxicity, as was sometimes observed for the non-target control, luciferase, at concentrations greater than 200 ng/μL. Implementing this pre-established dsRNA regime for laboratory screening of ACPs for RNAi will enable high-throughput discovery pipelines to identify effective dsRNA targets and, potentially, can aid in the evaluation of diverse RNAi biopesticide formulations for field application [8,20] at a minimal relative cost per treatment. Establishing a working threshold can also prevent the inadvertent use of excessive amounts of dsRNA for screening, which is expensive, can negatively influence RNAi efficiency [72], and may trigger off-target effects. This new knowledge also provides guidance for evaluating the effects of RNAi on selected non-target species that may be exposed to dsRNA, such as beneficial or benign arthropods and other vulnerable community organisms for which such data will be required prior to registration and deployment of RNAi biopesticides [31].

## Figures and Tables

**Table 1:** Primer sequences used for conventional amplification of psyllid target genes and for real-time, quantitative reverse transcriptase polymerase chain reaction (qRT-PCR) amplification of psyllid transcripts.

dsRNA Primers	Target Gene	Sequence (5′ to 3′)	Tm	
vATPase-A F	*vATPase-A*	TTATCTCAGAGCATTTACATCCCCAAGGGTG	61.4	
vATPase-A R		CTTGACAAGTGTATTCTCATGAACAAGACC	58.1	
Snf7 F	*Snf7*	GCTCACAAGCACATGGACGTGAACC	62.5	
Snf7 R		AACTTCAGTAGATCCTTGTCCAGTTCTTCC	59.6	
CHC F	*CHC*	CAGTACGCGGACGTGGAAGGAGG	63.0	
CHC R		TCGAAATAGCCGCGGTCCTGG	61.1	
**qRT-PCR Primers and Probes**	**Target Gene**	**Sequence (5′ to 3′)**	**Tm**	**Efficiency**
vATPase-A qF	*vATPase-A*	AGTGGTTATCCTGCCTACCT	53.7	95%
vATPase-A qR		CGTGCTGGCAGAGTCAAATGCTTG	61.1	
Probe		GGAGATACAGCACCCACAATAC	54.7	
Snf7 qF	*Snf7*	CAGCAGATTGATGGCACATTG	54.5	92%
Snf7 qR		GGCATTCTTCATGGTGGTAAGA	60.7	
Probe		AATTGAGATGCAGCGGGAAGCTCT	54.5	
CHC qF	*CHC*	AGCGAGGAGTTCCGTTTG	54.1	90%
CHC qR		GGTCCTGGTAGTAGTTGATGAG	59.6	
Probe		CGTGGTGCACGCAGATGAACTA	53.5	
RPL5 qF	*RPL5*	TCCAAAGGCAAGATCCAGAAA	53.7	93.5%
RPL5 qR		AGAAGCTCACTTTGGCTCAACGGA	60.5	
Probe		GGAAGTTAGCTTTGGCAGTAGA	54.2	

## Data Availability

Data are available from the authors upon request.

## Supplementary Materials

The following supporting information can be downloaded at: https://www.mdpi.com/article/10.3390/insects15010058/s1, Figure S1: Mortality of *Diaphorina citri* adults after 48 h dsRNA ingestion-access period (IAP) at concentrations of 10, 50, 100, 200, and 500 ng/µL on 20% sucrose solution, followed by 5 days post-IAP on orange jasmine, *Murraya* paniculata (L.) shoots. The RNase-free water and dsRNA-luciferase both with 20% sucrose were included as the non-treatment and non-target control, respectively. (A) dsRNA-clathrin heavy chain (*CHC*), (B) dsRNA-vacuolar ATPase subunit A (*vATPase-A*), (C) dsRNA-sucrose non-fermenting protein 7 (*Snf7*). Error bars represent the standard error of means (SEM). There was no statistically significant difference in psyllid mortality between treatments (ANOVA with Tukey’s HSD test, *p*-value > 0.05).
