# Supplementary figures and images for: Optimal dsRNA Concentration for RNA Interference in Asian Citrus Psyllid
Source: Insects. 2024 Jan 12;15(1):58. doi: 10.3390/insects15010058 (PMC10816725; doi:10.3390/insects15010058)

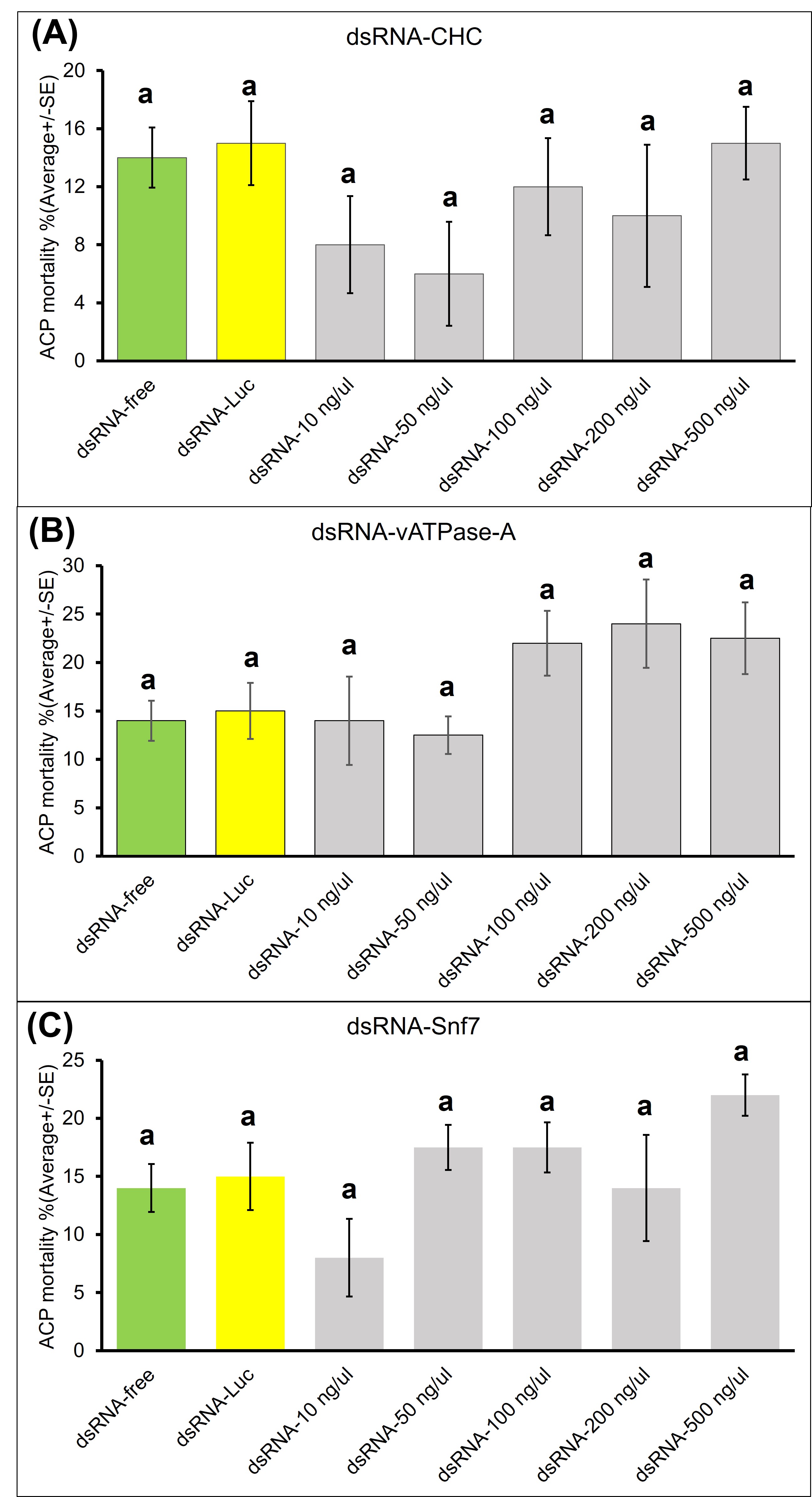

Supplement: Supplementary file 1 [file insects-15-00058-s001.zip › insects-2730265-Supplementary Figure S1.jpg]
